# EFFECTS OF CHILDCARE, WORK, AND CAREGIVING INTENSITY ON MALE AND FEMALE FAMILY CAREGIVERS

**DOI:** 10.1093/geroni/igad104.2644

**Published:** 2023-12-21

**Authors:** Hansol Kim, Scott Beach, Esther Friedman, Heidi Donovan, Richard Schulz

**Affiliations:** University of Pittsburgh, Pittsburgh, Pennsylvania, United States; University of Pittsburgh, Pittsburgh, Pennsylvania, United States; University of Michigan, Ann Arbor, Michigan, United States; University of Pittsburgh, Pittsburgh, Pennsylvania, United States; University of Pittsburgh, Pittsburgh, Pennsylvania, United States

## Abstract

**Objectives:**

The Behavioral Risk Factor Surveillance System sampled 54,076 caregivers between 2015 and 2017 providing an opportunity to evaluate risk factors for poor mental and physical health among a representative sample of U.S. adult caregivers. This study aimed to evaluate the impact of childcare, work status, and intensity of caregiving among men and women caring for older adults (n = 17,271).

**Methods:**

Controlling for sociodemographic factors, separate logistic regression analysis for women and men were carried out to assess the main and interaction effects of childcare, work status, and intensity of caregiving on number of poor mental and physical health days in the last month.

**Results:**

Intensive caregiving demands had adverse effects on both women and men, but being in the workforce was beneficial to both men and women. Women with children at home reported adverse mental health effects but better physical health, while men with children at home reported adverse physical health effects. For women, the combination of not working, children in the household, and high-intensity caregiving were most detrimental to their mental health. Among men, those not working with children in the household, regardless of caregiving intensity, were at highest risk of adverse mental health effects.

**Discussion:**

Our findings identify caregivers at high risk of adverse outcomes but also point to the need for more fine-grained analyses of how families negotiate the allocation of childcare, work, and caregiving responsibilities over time.

